# Donor Allospecific CD44^high^ Central Memory T Cells Have Decreased Ability to Mediate Graft-vs.-Host Disease

**DOI:** 10.3389/fimmu.2019.00624

**Published:** 2019-04-02

**Authors:** Wei Huang, Wenjian Mo, Jieling Jiang, Nelson J. Chao, Benny J. Chen

**Affiliations:** ^1^Division of Hematologic Malignancies and Cellular Therapy, Duke University Medical Center, Durham, NC, United States; ^2^Department of Hematology, School of Medicine, Guangzhou First People's Hospital, South China University of Technology, Guangzhou, China; ^3^Department of Hematology, Shanghai General Hospital Affiliated to Shanghai Jiao Tong University, Shanghai, China; ^4^Duke Cancer Institute, Duke University Medical Center, Durham, NC, United States

**Keywords:** alloreactive memory T cells, T_CM_, GVHD, skin graft rejection, OT-II, OVA

## Abstract

Data from both animal models and humans have demonstrated that effector memory T cells (T_EM_) and central memory T cells (T_CM_) from unprimed donors have decreased ability to induce graft-vs-host disease (GVHD). Allospecific T_EM_ from primed donors do not mediate GVHD. However, the potential of alloreactive T_CM_ to induce GVHD is not clear. In this study, we sought to answer this question using a novel GVHD model induced by T cell receptor (TCR) transgenic OT-II T cells. Separated from OT-II mice immunized with OVA protein 8 weeks earlier, the allospecific CD44^high^ T_CM_ were able to mediate skin graft rejection after transfer to naive mice, yet had dramatically decreased ability to induce GVHD. We also found that these allospecific CD44^high^ T_CM_ persisted in GVHD target organs for more than 30 days post-transplantation, while the expansion of these cells was dramatically decreased during GVHD, suggesting an anergic or exhausted state. These observations provide insights into how allospecific CD4^+^ T_CM_ respond to alloantigen during GVHD and underscore the fundamental difference of alloresponses mediated by allospecific T_CM_ in graft rejection and GVHD settings.

## Introduction

Graft-vs.-host disease (GVHD) is a major complication of allogeneic hematopoietic stem cell transplantation caused by alloreactive donor T cells (1). After bone marrow transplantation, the alloreactive donor T cells recognize the alloantigens presented by MHC in the recipients, and initiate the pathogenesis of GVHD. The contribution of different subsets of donor T cells to GVHD is different (2). T cells can be further separated into naive and memory T cells according to the expression of the cell-trafficking molecule CD62L and T cells activation molecule CD44. It has been proven that naive T cells, with the phenotype CD62L^+^ CD44^−^, have the strongest ability to induce vigorous GVHD in MHC-mismatch murine models. On the contrary, the memory T cells, including effector memory T cells (T_EM_, CD62L^−^ CD44^+^) and central memory T cells (T_CM_, CD62L^+^ CD44^+^) from either untreated or allo-antigen primed donors, do not cause GVHD or cause only minor GVHD after transplantation (3–6). Specifically, we have previously identified a population of T_CM_ that express high level of CD44 do not induce GVHD (5).

It has previously been reported that common virus specific memory T cells including EBV-specific and CMV-specific memory T cells do not GVHD in humans (7–11). However, since alloreactive memory T cells can be generated either by cross-reaction or allospecific memory reaction, it is important to further understand the biology and pathogenesis of the true allospecific memory T cells in GVHD. In the previous research, we used an antigen-specific murine model to study allospecific T_EM_ in GVHD (12). By transferring the naive TEa cells into Rag-1^−/−^ mice following by *in vivo* priming with splenocytes from CB6F1 (H2^b^/I-E^+^ strain), T_EM_ cells from the primed animals maintained the memory function to mediate skin graft rejection, but did not mediate GVHD when transplanted into lethally irradiated CB6F1 hosts. However, allospecific T_CM_ population could not be generated in this model. To study the potential of alloreactive TCM to induce GVHD, we utilized a novel GVHD model induced by T cell receptor (TCR) transgenic OT-II T cells. Using this model, we were able to generate antigen-specific T_CM_ by immunizing donor mice directly and further demonstrated that these cells mediated secondary skin graft rejection while did not induce GVHD.

## Materials and Methods

### Mice

C57BL/6 mice were purchased from The Jackson Laboratory (Bar Harbor, ME). B6.Cg-Tg(TcraTcrb)425Cbn/J (OT-II) mice and C57BL/6-Tg(CAG-OVA)916Jen/J (OVA) mice (13) were purchased from The Jackson Laboratory as breeders, and were bred and maintained at Duke University in a specific pathogen-free facility during the study. To enable cell tracing, OT-II mice were further crossed with GFP^+^ mice and Luciferase^+^ mice (a generous gift from Dr. Andreas Beilhack and Dr. Robert Negrin, Stanford University) to generate OT-II^+^ Luciferase^+^ GFP^+^ triple positive mice. For all the strains, both female and male mice were used in this study. The donor mice were primed at 6–8 weeks old. The recipient mice were between 7 and 16 weeks old at the time of transplantation. All animal care and experimental procedures were approved by National Institute of Health and Duke University Institutional Animal Care and Use Committee.

### Generation of Allospecific T Cells

To generate allospecific OT-II memory T cells *in vivo*, OT-II mice between the age of 6–8 weeks were immunized with OVA protein (Sigma-Aldrich, MO, USA) emulsified in complete Freund's adjuvant (Sigma-Aldrich, MO, USA) i.p. at 100 ug/mouse (14). Mice were then hosted in a pathogen-free facility for 8 weeks before use.

### T-Cell Depletion From Bone Marrow

OVA mice between age 7–16 weeks were used as T-cell depleted (TCD) bone marrow donors. T cells were depleted from bone marrow using anti-CD90.2 antibody and complement as previously published. In brief, bone marrow cells were flushed out from the long bones of donor mice and strained through a 70 μm cell strainer (Becton Dickinson labware, NJ, USA). Cells were then resuspended in cytotoxicity medium, incubated with anti-CD90.2 monoclonal antibody (clone 30H12; BD Pharmingen, CA USA) at 4°C for 1 h. The cells were washed once and then resuspended in cytotoxicity medium containing 1:10 Low-Tox-M Rabbit Complement (Cedarlane, Canada). The cells were then incubated at 37°C for 60 min and washed twice before use.

### T Cell Separation

**Figure 1:**
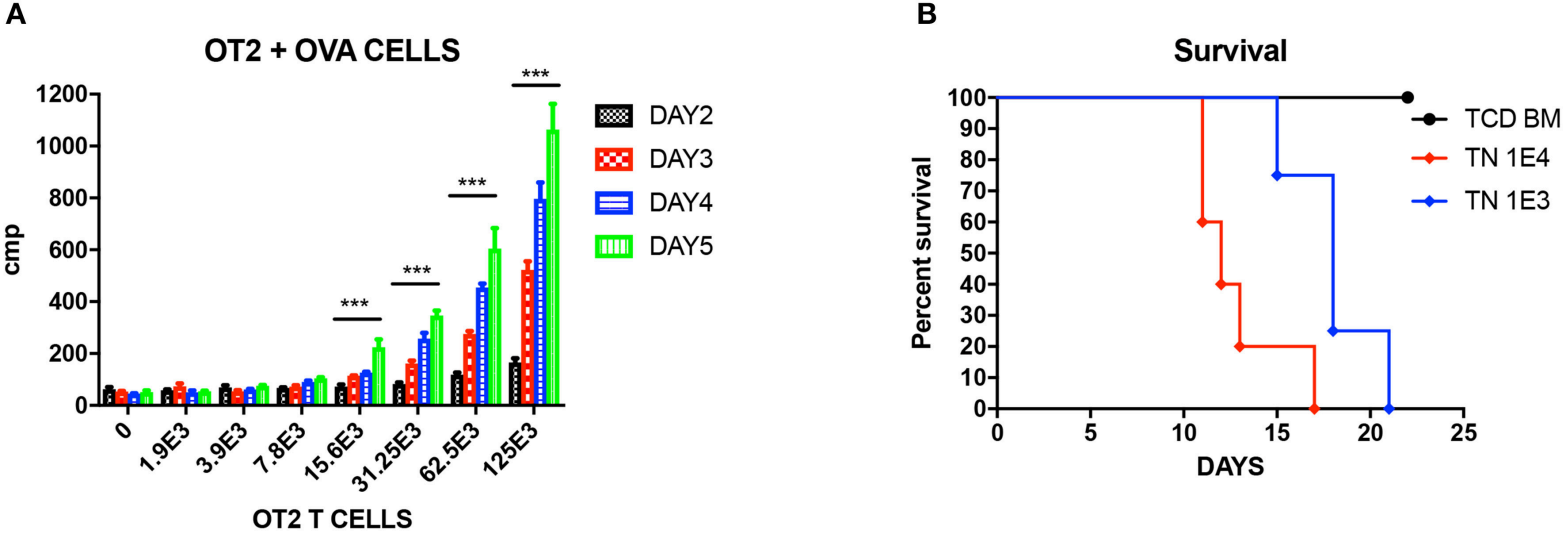
Unprimed OT-II T cells reacting to OVA cells. **(A)** Mixed lymphocyte reaction (MLR) of unprimed OT-II T cells cultured with different doses of OVA splenocytes, cultured for different days. Three wells each condition. Experiment repeated twice. ^***^P < 0.001 for four titrations. Analyzed using multiple *t* test. **(B)** Titration of unprimed sorted T_N_ from OT-II mice and injected into OVA mice to induce GVHD. *P* < 0.01 for both doses compared to TCD BM. *N* = 5 each group. Experiment repeated twice.

OT-II mice primed for 8 weeks were used as T-cell donors. Purified T cells were separated from splenocytes using mouse Pan T Cell Isolation Kit II (Miltenyi, Germany). The purified T cells were then stained with APC–conjugated anti-CD62L (clone MEL-14), PE-conjugated anti-CD4 (clone CT-CD4), PerCy5.5-conjugated anti-CD44 (clone IM7) from BD PharmMingen (CA, USA), and sorted into different T-cell subsets according to Figure 1 Panel using MoFlo Astrios Cell Sorter (Backman Coulter, IN, USA). Sorted cells were washed and counted before use. The purity after sorting was 92–96% for T_N_, 90~95% for T_EM_ with 2–3% T_CM_ contamination, and 86~92% for T_CM_ with 2~9% T_EM_ contamination and 1–2% T_N_ contamination.

### Mixed Lymphocyte Reaction (MLR)

The proliferation assay was performed as described previously (5). Graded numbers of purified OT-II T cells as indicated were plated in 96-wells, flat-bottomed culture plates with 5 × 10^5^ irradiated (20Gy) OVA splenocytes in a final volume of 200 μl. After incubation at 37°C in 5% CO_2_ for a specified period as indicated, cultures were pulsed with ^3^H-thymidine (1μCi [0.037MBq]/well). Cells were harvested after another 16 h of incubation, and counted in a MicroBeta Trilux liquid scintillation counter (EG&G Wallac, Turku, Finland). Triplicate cultures were set up for each cell population tested.

### GVHD Model

OVA mice were lethally irradiated (10.5 Gy) using Cs irradiator and injected with 1 × 10^7^ TCD BM and different numbers of purified OT-II cells through tail vein. Survival and clinical scores of GVHD including body weight change, fur ruffling, skin changes, hunching posture, diarrhea, and activity were monitored daily. Moribund mice were sacrificed according to protocol approved by the Duke University Institutional Animal Care and Use Committee.

### Skin Transplantation

The skin transplantation protocol was modified as previously published (12). In brief, tail skin from OVA mice was removed from sacrificed donors, cut into ~0.5 × 0.5 cm^2^ pieces, and kept on swab damped with cold PBS. The C57BL/6 recipient mice were anesthetized with isoflurane (Halocarbon, GA, USA) with the right lumbar region shaved and sanitized with iodine solution followed by alcohol. A graft bed was prepared by removing an area of skin down to the level of the intrinsic muscle using fine scissors. The graft was fitted to the prepared bed, sutured with 5-0 surgical suture, and wrapped with an adhesive plastic bandage. The bandage was removed 4 days after surgery. Skin graft survival was assessed everyday by visual and caliper measuring. Rejection was defined as the first day when the entire epidermal surface area of the graft was <10% of original.

### Bioluminescent Imaging

Mice were monitored for T-cell tracking once per week after bone marrow transplantation. For *in vivo* imaging, mice were anesthetized with isoflurane and injected intraperitoneally with 50 mg/kg D-Luciferin (PerkinElmer, CT, USA) 10 min before imaging with a Xenogen IVIS 100 imaging system (Xenogen Corporation, Alameda, CA, USA) at maximum signal intensity using 5 min exposure time. Regions of Interest (ROIs) were drawn using Living Image 2.5 software (Caliper, MA, USA).

### Flow Cytomery Analysis

Single cell suspension of splenocytes were prepared as described before (5, 12). In brief, organs were removed from the sacrificed mice, and gently crunched using the gridded end of a syringe on a 70 μm cell strainer. Cells were then strained, treated with red blood cell lysis, washed, and stained with antibodies for flow cytometry per manufacturer's protocol. The antibodies used were as follow: PE anti-mouse Vα2 TCR (B20.1), PE/Cy7 anti-mouse CD62L (MEL-14), APC anti-mouse CD4 (RM4-5), PerCP-Cy5.5™ CD44 (IM7) (all from BD Biosciences, CA, USA). Flow cytometry was performed using a BD FACSCanto (BD Biosciences). Data were analyzed with BD FACSDiva™ Software (BD Biosciences).

### Statistical Analysis

Statistical analysis was performed using Prism GraphPad (GraphPad Software, CA, USA) and Excel (Microsoft, WA, USA). For survival studies, log-rank Mantel-Cox test was used. For MLR, body weight changes, GVHD score, and bioluminescent measurement, Student's *t* test, multiple *t* test, and multi-way ANOVA test were used. Level of significance was set at *P* < 0.05. Bar graphs represent mean ± SEM.

## Results

### Unprimed OT-II T Cells React to OVA Cells

We first tested the reactivity of OT-II T cells again OVA cells in vitro. Unprimed OT-II T_N_ were sorted from OT-II mice as responding cells, and cocultured with 5 × 10^5^ lethally irradiated OVA splenocytes as stimulators at graded ratio for different time period from 2 to 5 days. By analyzing the ^3^H-thymidine uptakes, it is shown that from T cells: OVA splenocytes 1:10 on, OT-II unprimed T_N_ can be efficiently stimulated and proliferated (Figure 1A. P < 0.01). When we titrated these unprimed T_N_ into lethally irradiated OVA mice, we proved that unprimed T_N_ could cause lethal GVHD at a low dose of 1,000 cells, and the GVHD effect was dose dependent (Figure 1B).

### Generation of Functional OVA Antigen-Specific OT-II Memory T Cells

**Figure 2:**
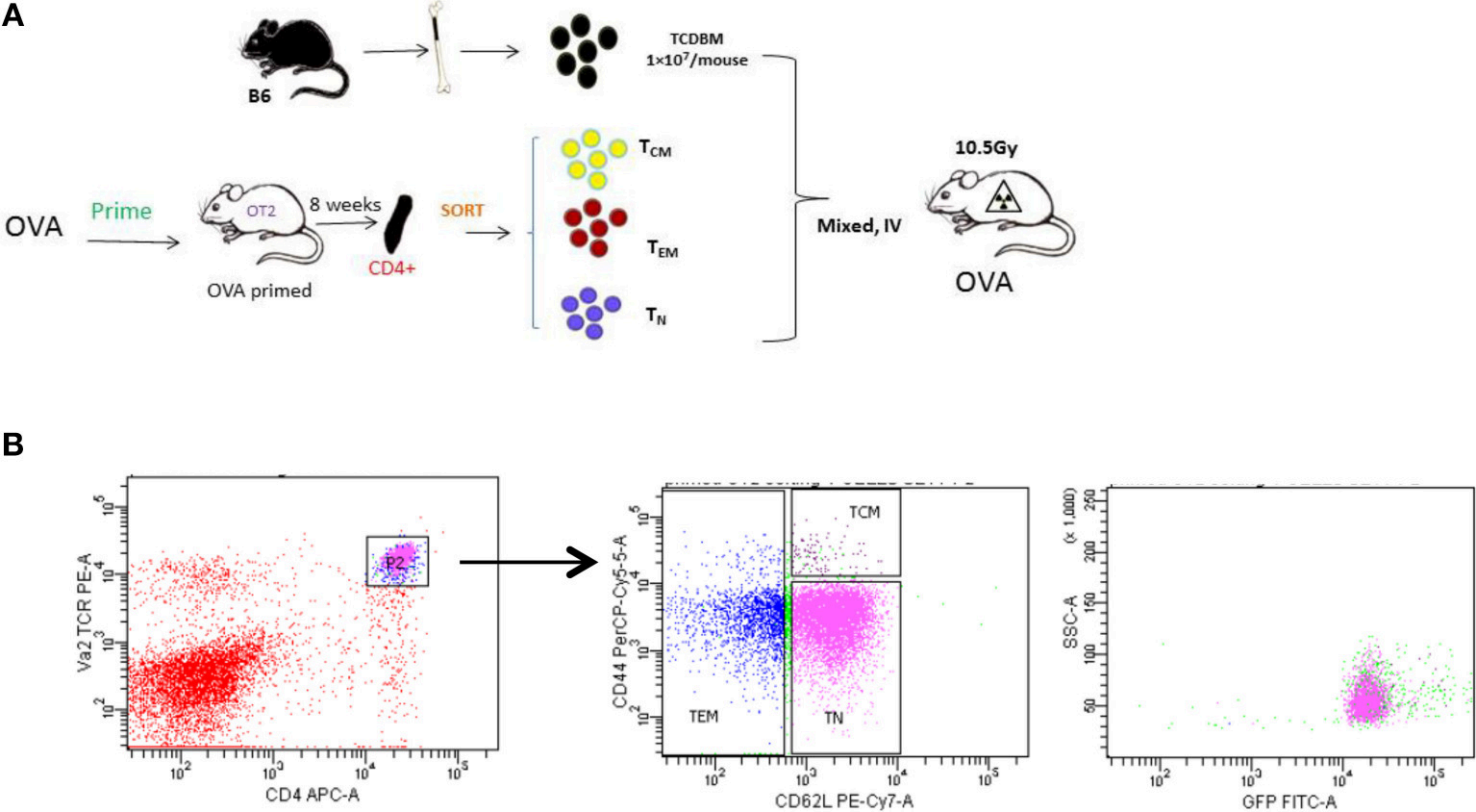
Generating allospecifc T_CM_ cells. **(A)** A schematic for antigen-specific T-cell generation. OT-II transgenic mice were primed with OVA peptide 8 weeks before transplantation. Primed OT-II transgenic T cells were sorted into three subsets (T_N_, T_CM_, T_EM_) and transplanted into lethally irradiated OVA mice at the dose of 1 × 10^3^ cells/mouse, along with 1 × 10^7^ T-cell-depleted BM cells. **(B)** Gating of antigen-specific T cells. Flow cytometry analysis was performed in OT-II mice at least 8 weeks after priming. All OT-II T cells were GFP positive.

In order to study the role of antigen-specific central memory T cells in GVHD, we first generated a T-cell mediated antigen-specific GVHD model using the OT-II/OVA system as showed in Figure 2A. We first immunized the OT-II donor mice by injecting emulsified OVA protein intraperitoneally and housed the mice for 8 weeks to generate OVA-specific memory OT-II cells. OT-II T cells as identified as CD4^+^ Vα2^+^ cells were sorted into naive (T_N_, CD62L^+^ CD44^low^), effector memory (T_EM_, CD62L^−^), and central memory (T_CM_, CD62^+^CD44^high^) T cell subsets. Flow cytometry also confirmed that all the sorted antigen specific cells are GFP^+^ (Figure 2B).

**Figure 3:**
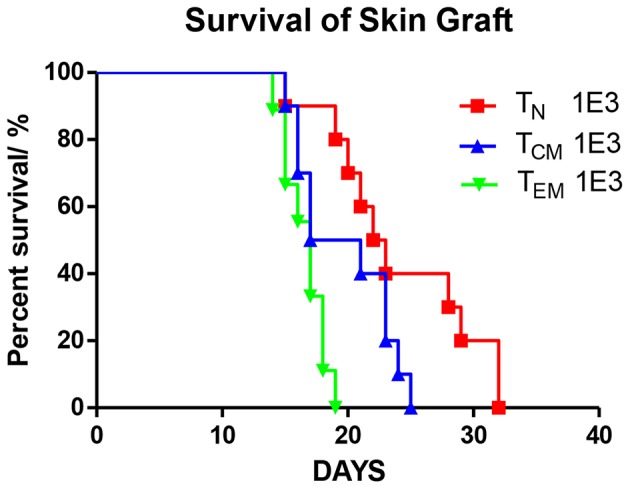
Allospecific T_CM_ mediate secondary skin graft rejection. OT-II T cells were sorted into three subsets (T_N_, T_CM_, T_EM_) after 8 weeks priming. 1 × 10^3^ T cells of each subset were transplanted into C57BL/6 female mice which were transplanted with OVA tail skin graft on the subsequent day. Graft survival was observed daily. *P* = 0.0002, T_EM_ recipients vs. T_N_ recipients. *P* = 0.078, T_CM_ vs. T_N_. *n* = 10 for each group. Data were pooled from two independent experiments.

To verify the immune function of these immunized OT-II cells, we introduced the secondary skin graft rejection model. C57BL/6 mice were transferred with 1 × 10^3^ OT-II T_N_, T_CM_, or T_EM_. On the subsequent day, a 0.5 × 0.5 cm^2^ piece of tail skin peeled from OVA mice were transplanted to the right lumbar region of the recipients (Figure 3). By measuring the area of the live graft daily, we demonstrated that the graft survival times in mice that received T_EM_ or T_CM_ injections were significantly shorter (*P* = 0.0002) or trended to be shorter (*P* = 0.078) compared to that in T_N_ recipients These data are consistent with the previous publications (2, 4, 12) and indicate that the different OT-II subsets sorted from OVA-immunized OT-II mice were functionally anti-OVA memory T cells.

### Antigen-Specific T_EM_ and T_CM_ Do Not Cause GVHD

**Figure 4:**
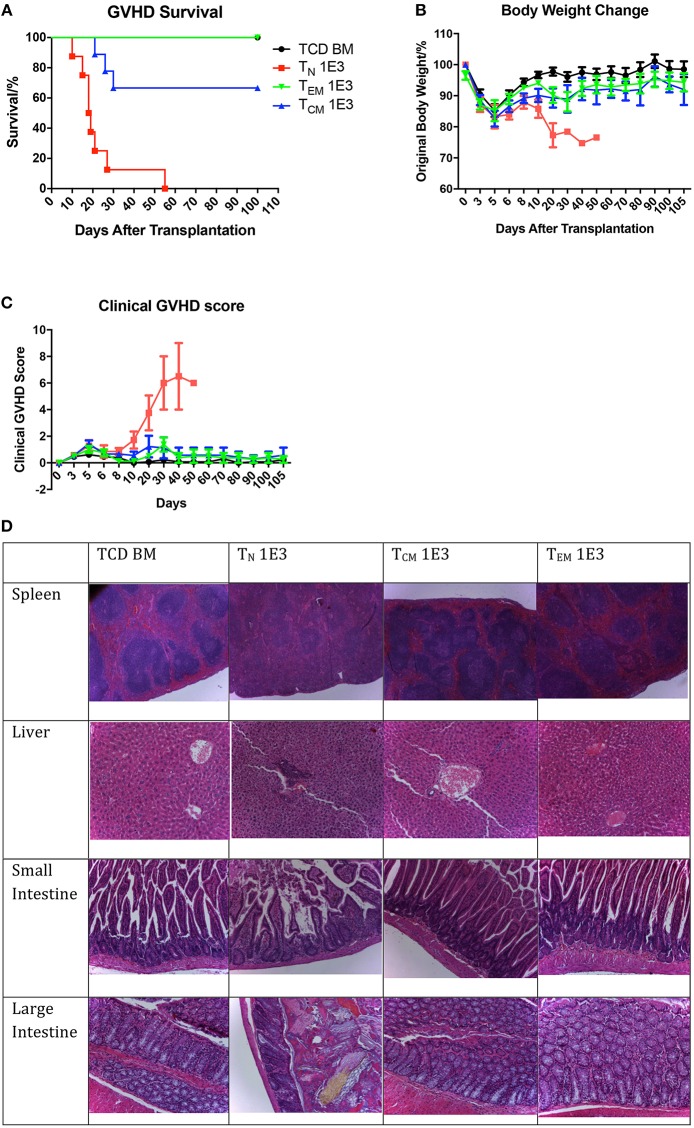
Allospecific T_CM_ have decreased ability to induce GVHD. Primed OT-II T cells were sorted into three subsets and transplanted into lethally irradiated OVA mice at the dose of 1 × 10^3^ along with 1 × 10^7^ TCD BM. Mice survival, body weight, and GVHD scores (body weight, posture, activity, fur, skin integrity, diarrhea) were monitored daily. *n* = 9 for each group. Data pooled from two independent experiments. **(A)** T_EM_ and T_CM_ recipients have better survival comparing to T_N_ recipients. *P* < 0.0001, T_N_ vs. TCD BM. *P* = 0.065, T_CM_ vs. TCD BM. Estimate hazard ratio between T_N_ and T_CM_ is 7.4821. **(B)** T_EM_ and T_CM_ recipients have better body weight recovery comparing to T_N_ recipients. *P* < 0.0001, T_N_ vs. TCD BM. *P* = 0.043, T_CM_ vs. TCD BM. *P* = 0.1136, T_EM_ vs. TCD BM. **(C)** T_EM_ and T_CM_ recipients have lower GVHD score comparing to T_N_ recipients. *P* < 0.001, T_N_ vs. TCD BM. *P* = 0.0937, T_CM_ vs. TCD BM. *P* = 0.5324, T_EM_ vs. TCD BM. *P* < 0.001, T_N_ vs. T_CM_ and T_EM_. **(D)** Histology on GVHD target organs. In T_N_ recipients, GVHD pathological changes can be found in spleen as fibrosis and hypocellularity, in liver as portal triad lymphocyte infiltration with bile duct injury and cholangitis, in intestines as crype/gland destruction with epithelial cell apoptosis and lymphocyte infiltration. TCD BM, T_CM_, and T_EM_ recipients have relatively normal organ morphology.

After confirming the anti-OVA function of the memory OT-II T cells, we tested these cells in the OT-II anti-OVA antigen specific GVHD model by injecting 1 × 10^3^ sorted T_N_, T_CM_, or T_EM_ subsets of OT-II T cells from OVA-primed OT-II mice together with 1 × 10^7^ TCD-BM from OVA mice into lethally irradiated OVA recipients. The survival, body weight changes, and GVHD clinical score were monitored daily. Unlike what was observed in skin rejection model, the mice that received T_N_ cells had the earliest death related to GVHD, with all the mice died within 56 days, while mice that received T_EM_ had 100% survival over 100 days, and mice that received T_CM_ had 70% survival till 100 days (Figure 4A). As to body weight recovery and GVHD clinical score, T_N_ recipients had the worst performance compared to mice that received memory T cells. Mice receiving either T_EM_ or T_CM_ had similar recovery status compared to TCD BM mice, which were the negative controls (Figures 4B,C). Using higher T cell dose at 1 × 10^4^ for all cell types led to similar conclusion (Supplemental Figure 1). These result indicate that, although antigen-specific T_CM_ cells result in some mortality in acute phase, neither T_EM_ nor T_CM_ cause significantly clinical GVHD in the survivors in long-term follow-up.

To verify this, we further collected the target organs of GVHD including spleen, liver, small and large intestines, when sacrificing the mice because of morbidity or at Day 28, and accessed for histopathological changes (Figure 4D). In the organs from TCD BM mice, the histological structure of the organs was clear with cells well aligned. However, in the organs from T_N_ mice, significant GVHD histological structure changes were seen, including the blurred edges between the white pulps and red pulps in the spleen, portal vein thrombosis and lymphocyte infiltration in the liver, disruptions of the villi and crypts with lost of epithelial cells and goblet cells in the small and large intestines. These pathological changes in the organs were not presented in the organs from mice received T_EM_ or T_CM_ cells. The histological results further confirmed that although OT-II antigen-specific memory T cells had the memory function to reject OVA-expressed skin grafts faster compared to T_N_ cells, neither T_EM_ nor T_CM_ caused histopathological GVHD changes in the GVHD target organs.

### Antigen-Specific T_EM_ and T_CM_ Proliferated Less but Persisted in GVHD Hosts

**Figure 5:**
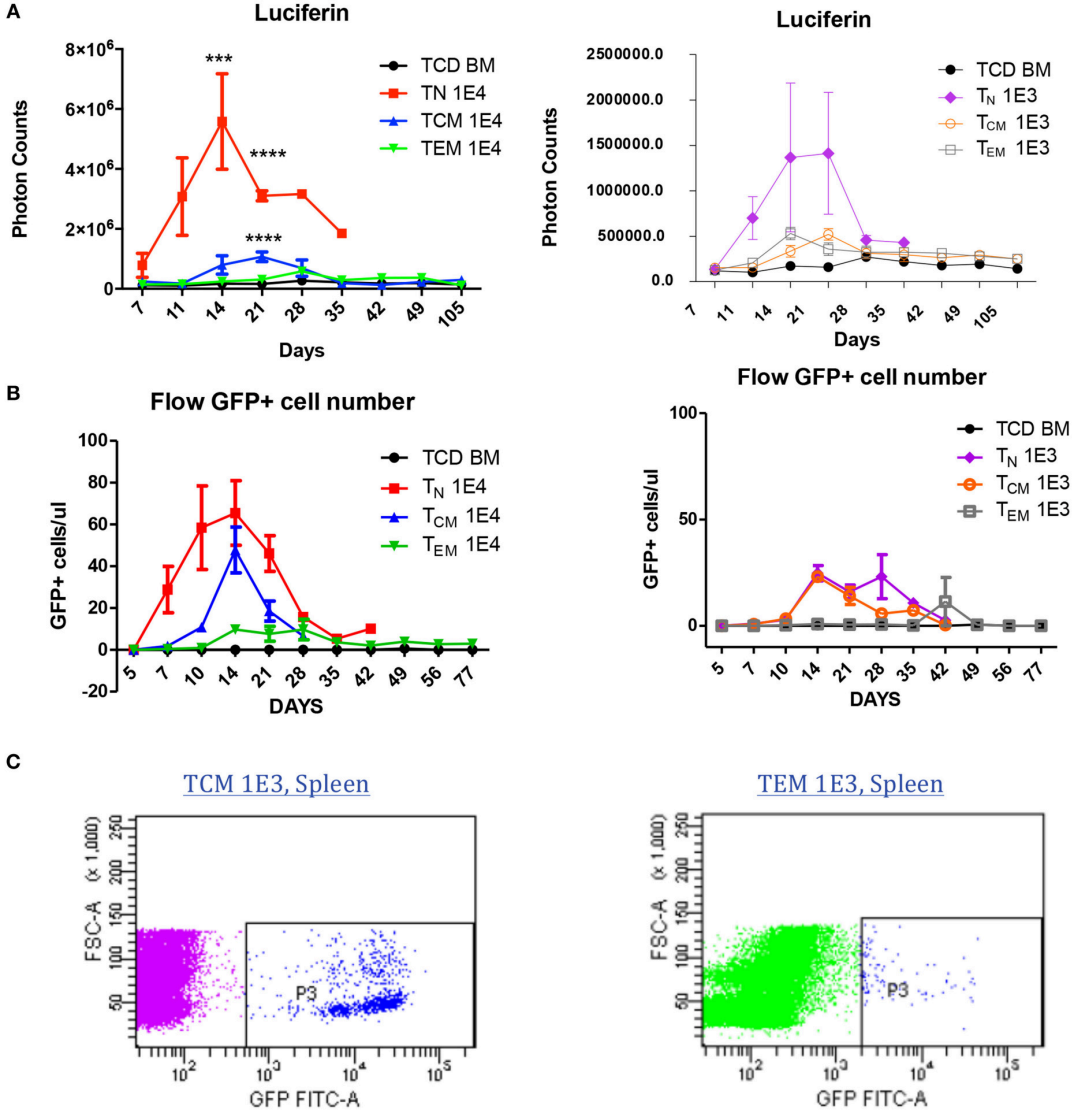
Allospecific T_CM_ are exhausted in GVHD model. Luciferase and GFP labeled OT-II T cells were primed and transplanted into OVA mice at two doses (1 × 10^4^, 1 × 10^3^). *n* = 7 each group. Experiments repeated twice. **(A)** Significant T-cell proliferation was observed in T_N_ recipients, but not in T_CM_ and T_EM_ groups using luciferin tracing. Luciferin tracing were performed on day 7, 11, 14 after transplantation and once every week. **(B)** GFP^+^ labeled OT-II T cells in peripheral blood were significantly increased in T_N_ and T_CM_ groups but not in T_EM_ group. Peripheral blood was collected from transplanted OVA mice (*n* = 4) on day 5, 7, 10, 14 after transplantation and once every week. GFP^+^ cells were counted using flow cytometry. The different pattern of T-cell proliferation between **(A)** and **(B)** may be caused by T-cell distribution in the body. **(C)** T cells were detectable in GVHD survivors' organs. ^***^P < 0.001; ^****^P < 0.0001, compared with TCD BM.

To understand why antigen-specific memory T cells did not cause GVHD, we used bioluminescent imaging (BLI) and flow cytometry to track the antigen-specific T cell expansion *in vivo* after BMT. To generate GFP- and luciferase-expressing OT-II T cells, OT-II mice were crossed with Luciferase-reporter mice, and further crossed with GFP positive mice. Cells were sorted as described in Figure 1 and transplanted into irradiated OVA mice at two different cell doses to enable cell tracing. BLI revealed that, at both cell doses, T_N_ recipients had a much higher number of photon counts compared to either T_EM_ or T_CM_ recipients, indicating the robust expansion of T_N_ after BMT. T_CM_ recipients also had a higher T-cell signal on Day 21 compared to T_EM_ recipients, but soon declined to a comparable low level after 28 days (Figure 5A). The GFP^+^ cell number in the peripheral blood detected by flow cytometry also showed the same trend. Similar but different to BLI, in the peripheral blood, GFP^+^ OT-II T_CM_ cells had the peak around Day 14 and started to decline afterwards (Figure 5B). Although the mice receiving the lower dose of T cells did not have detectable significant expansion peak due to limited cell numbers and technical sensitivity, the same trend detected in both methods using different cell doses indicated the robust expansion of T_N_, the transient expansion of T_CM_, and the limited expansion of T_EM_ in an antigen specific GVHD model. Since the detected number of T_EM_ cells were very limited in both methods, we further confirmed the existence of the GFP cells in the spleens of T_EM_ and T_CM_ recipients 30 days after BMT using flow cytometry (Figure 5C). Although the limited cell number in the recipients prevented us to further analyze the cell surface markers for mechanistic studies, the existence of GFP^+^ antigen-specific memory T cells inside the target organs without causing GVHD suggested the exhausted status of these cells in GVHD model.

## Discussion

In our study, we successfully utilized the OT-II / OVA system to generate allo-specific T_EM_ and T_CM_ by directly immunizing donor mice with alloantigens. These T_EM_ and T_CM_ were phenotypically the same as those isolated from polyclonal mice (5). We further confirmed that T_EM_ and T_CM_ separated from primed OT-II mice were functionally memory T cells because they rejected second-set skin grafts faster than T_N_ did. By transplanting these different subsets of primed T cells into OVA mice, we proved that OT-II T_N_ cells mediated the vigorous GVHD, while T_EM_ did not cause GVHD in OVA mice. Although primed OT-II T_CM_ resulted in some death within the first 3 weeks, the survival rate was still significantly higher than T_N_ group, while the body weight recovery, GVHD score, and histological changes in the target organs were all similar to T_EM_ recipients, indicating that T_CM_ do not cause or cause very minor GVHD. These results are consistent with the previously published data demonstrating that alloreactive T_EM_ and T_CM_ would not cause GVHD (3–6, 12, 15). Our finding further verified this conclusion under the antigen-specific condition with no interference of antigen cross-presentation.

Our study has the important clinical significance in T-cell therapy in BMT patients. Antigen-specific T cells against host antigen are believed to be the major players in inducing GVHD. In our study, we demonstrated that not only antigen-specific T_EM_ but also T_CM_ against host antigen do not cause GVHD. Currently, naive T cells depletion and anti-virus memory T cells transfusion are under clinical trial for BMT patients to preserve T-cell anti-infection function while preventing GVHD (11, 16–20). Similar studies are also under investigation using tumor specific T cells (21–24). Our study further supports the safety and feasibility of naive T cell depletion and using virus- and tumor-specific memory T cells to prevent infections and tumor relapses for BMT patients without causing GVHD.

One major difference between human and mouse memory T cells is that human memory T cells may contain true alloantigen specific T cells while those from normal mice do not. In humans, alloantigen specific memory T cells are generated when naïve T cells are exposed to alloantigens during transfusion or pregnancy (25). Even though multiple groups have demonstrated in several different animal models that memory T cells do not induce GVHD, (3–6, 15) one major concern when translating these findings into clinic is that human memory T cells may behave differently because they contain true alloantigen specific T cells. The findings from the current study at least partially address this concern because we demonstrate that even true alloantigen specific T cells have decreased ability to induce GVHD.

We also further investigate the primed OT-II T cells proliferation and retention in the organ after transplantation. By using bioluminescent imaging, we proved that compared to T_N_ cells that underwent vigorous proliferation in the first 3 weeks, OT-II T_EM_ and T_CM_ had very limited proliferation in the spleens after transplantation. This finding was supported by Dr. Brede's research, and further advanced his findings (26). Compared to T_EM_ cells, T_CM_ had a more potent proliferation in the peripheral blood between Day 10 to Day 21. This may explain some of the GVHD related death in the first 3 weeks. When we further traced these cells, we found that even 30 days after the BMT, in the survivors' spleens we could still identify the retention of transplanted T_CM_ and T_EM_ cells. This result confirmed that the antigen specific memory T cells persist in the hosts after BMT but failed to induce GVHD, suggesting a potential status of T-cell exhaustion. Due to the limited number of T cells that we could recover from the recipient mice, we were unable to completely understand the mechanism by which alloreactive T_CM_ could reject skin graft but could not induce GVHD, and why the existing alloreactive T_CM_ remained for more than 30 days but did not induce GVHD. Hypothetically, we speculate that the fate of antigen-specific memory T cells would be different in the environment that encounters a small amount of removable antigens vs. the environment that is surrounded by a large amount of non-removable antigens. According to the previous research studying T-cell immunology in viral infections, (27) memory T cells were the dominant T-cell population in peripheral blood in acute viral infection when virus titer was low and the virus could be eliminated. On the contrary, in chronic viral infection when virus load was high and the virus sustained, naive T cells were the dominant T cells, and memory T cells had a limited clonal expansion within the first week compared to acute infection. These memory T cells were exhausted and underwent clonal depletion within 7–21 days (13, 28–31). The way that we challenged the alloreactive T-cell transplanted mice with skin graft was very similar to acute viral infection, while the BMT especially GVHD situation was very similar to chronic viral infection. This explains why the antigen-specific T_CM_ could reject skin graft, but could not induce GVHD later. We speculate that the long-term existing T_CM_ would get exhausted to a specific non-removable alloantigen in an GVHD setting.

In the previous studies, different TCR Tg T-cell of a single specificity models were used to study to alloreactive GVHD (3). These models include the CD8-mediated major MHC-mismatched 2C Tg model which is L^d^-specific, the CD4-mediated MHC-mismatched 3BBM74 model which is I-A^bm12^-specific and D10 model which is I-A^b^-specific, and the CD4-mediated miHAg-mismatched TEa model, and the CD4-mediated TS1 TCR Tg model which recognize the S1 epitope of HA on the HA104 Tg mice (3, 12, 32–36). In most of these studies, T cells were immunized and activated *in vivo* or *in vitro*, and transferred and expanded in RAG1^−/−^ mice. In these models, T-cell homeostasis is unpreventable, while the separation of T_CM_ is hard to achieve due to continually CD44 expression. In Juchem et al. study, the use of *in vitro* immunized TS1 cells injecting into HA mice, which was a single-antigen TCR Tg model, reached similar conclusion about T_EM_ with what we have seen in OT-II T-cell OVA host model (3). However, in the TS1-HA model, the T_CM_ could not be well distinguished from the T_N_ cells due to the continually CD44 expression, and mice receiving T_CM_ had shown signs of GVHD. Thinking that the high potent of T_N_ to cause GVHD, the different phenomenon of T_CM_ in TS1-HA model and in our model may be caused by the very small number of T_N_ contamination. In our OT-II-OVA model, due to a clearer separation of T_CM_ subsets, we were able to focus on the CD44^high^ expression population, and proved that antigen-specific T_CM_ did not cause GVHD.

Based on the previous findings from Strober's group demonstrating that memory CD4^+^ T cells do not directly mediate GVT effect by themselves (2), we do not expect OT-II T_CM_ are able to mediate direct GVT effect because they are CD4^+^ T cells. However, based on our finding that antigen-specific T_CM_ was able to reject OVA-expressing skin graft (Figure 3) and the ability of primed CD4 cells to facilitate tumor killing (37), we believe it is reasonable to speculate that these antigen specific T_CM_ maintain at least indirect GVT activity.

The OT-II into OVA murine model that we provided is novel to study the antigen-specific memory T cells in murine GVHD model. There are two major advantages that contributed to the study. First, in OT-II mice, the memory T cells can be directly generated and expanded *in vivo*, and further sorted into well differentiated subsets including T_N_, T_EM_, and T_CM_. This enables the study of single peptide antigen-specific subsets of T cells generated in a physical condition *in vivo*. Second, OVA is a commonly used labeled antigen on various cancer cell lines. OT-II / OVA GVHD model facilitates the study of the anti-tumor effect of different antigen-specific T-cell subsets in the GVHD model.

There are also some limitations and unanswered questions that need to be aware of. First, the antigen-specific model is very sensitive to T-cell number. Only 1,000 antigen-specific T_N_ cells can cause lethal GVHD, and 1,000 T_CM_ cells can partially cause GVHD. The limitation of small number of cells becomes the obstacle for further cell tracing and cellular and molecular mechanism study. Secondly, the model is still preliminary. Further information about the exhaustion markers and functional assays of the cells injected would be more helpful to define the status and biological characteristics of the antigen specific memory T cells in GVHD.

In conclusion, we have established a feasible antigen-specific TCR Tg GVHD model by immunizing OT-II mice *in vivo* to generate memory T cells, and transplanting these T cells into OVA mice to induce GVHD. We have demonstrated that antigen-specific T_EM_ and T_CM_ model do not cause GVHD due to a decreased proliferation potency after BMT, but can exist in the hosts' organs for long possibly due to exhaustion.

## Author Contributions

BJC and WH designed the experiments. WH, WM, JJ performed the experiments. WH did the statistic analysis. WH, BJC, and NJC wrote the manuscript.

### Conflict of Interest Statement

The authors declare that the research was conducted in the absence of any commercial or financial relationships that could be construed as a potential conflict of interest.

## Supplementary Material

Supplemental Figure 1 Alloreactive T_CM_ cells at higher dose have decreased ability to induce GVHD. Primed OT-II T cells were sorted into three subsets and transplanted into lethally irradiated OVA mice at the dose of 1 × 10^4^ along with 1 × 10^7^ TCD BM. Mice survival, body weight, and GVHD scores (body weight, posture, activity, fur, skin integrity, diarrhea) were monitored daily. **(A)** T_EM_ and T_CM_ recipients had better survival comparing to T_N_ recipients. *P* < 0.0001, T_N_ vs. TCD BM. *P* < 0.0001, T_CM_ vs. TCD BM. **(B)** T_EM_ and T_CM_ recipients had better body weight recovery comparing to T_N_ recipients. *P* < 0.0001, T_N_ vs. T_CM_ and T_EM_ on Day 8 and Day 10. **(C)** T_EM_ and T_CM_ recipients had lower GVHD score comparing to T_N_ recipients. *P* < 0.001, T_N_ vs. T_CM_ and T_EM_ on Day 8 and Day 10. *n* = 14–16 for each group. Data pooled from three independent experiments.
