# Supplementary figures and images for: Donor Allospecific CD44high Central Memory T Cells Have Decreased Ability to Mediate Graft-vs.-Host Disease
Source: Front Immunol. 2019 Apr 2;10:624. doi: 10.3389/fimmu.2019.00624 (PMC6454869; doi:10.3389/fimmu.2019.00624)

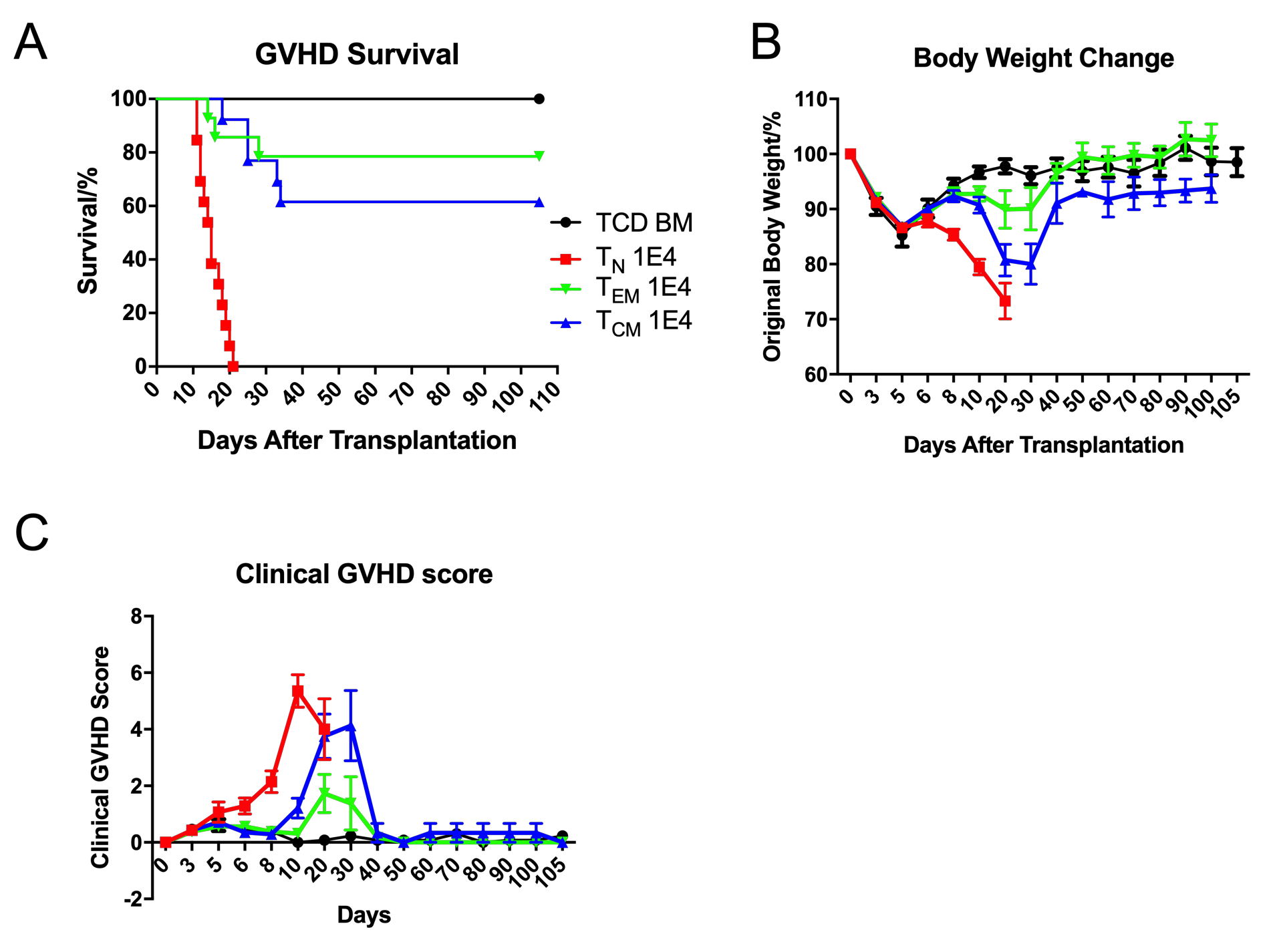

Supplement: Supplemental Figure 1 — Alloreactive TCM cells at higher dose have decreased ability to induce GVHD. Primed OT-II T cells were sorted into three subsets and transplanted into lethally irradiated OVA mice at the dose of 1 × 10^4^ along with 1 × 10^7^ TCD BM. Mice survival, body weight, and GVHD scores (body weight, posture, activity, fur, skin integrity, diarrhea) were monitored daily. (A) TEM and TCM recipients had better survival comparing to TN recipients. P < 0.0001, TN vs. TCD BM. P < 0.0001, TCM vs. TCD BM. (B) TEM and TCM recipients had better body weight recovery comparing to TN recipients. P < 0.0001, TN vs. TCM and TEM on Day 8 and Day 10. (C) TEM and TCM recipients had lower GVHD score comparing to TN recipients. P < 0.001, TN vs. TCM and TEM on Day 8 and Day 10. n = 14–16 for each group. Data pooled from three independent experiments. [file Image_1.TIFF]
